# Systematic Review of Presymptomatic Treatment for Spinal Muscular Atrophy

**DOI:** 10.3390/ijns10030056

**Published:** 2024-08-14

**Authors:** Katy Cooper, Gamze Nalbant, Anthea Sutton, Sue Harnan, Praveen Thokala, Jim Chilcott, Alisdair McNeill, Alice Bessey

**Affiliations:** School of Medicine and Population Health, University of Sheffield, Sheffield S1 4DA, UK; g.nalbant@sheffield.ac.uk (G.N.); a.sutton@sheffield.ac.uk (A.S.); s.harnan@sheffield.ac.uk (S.H.); p.thokala@sheffield.ac.uk (P.T.); j.b.chilcott@sheffield.ac.uk (J.C.); a.mcneill@sheffield.ac.uk (A.M.); a.r.bessey@sheffield.ac.uk (A.B.)

**Keywords:** systematic review, spinal muscular atrophy, presymptomatic treatment, efficacy of treatment

## Abstract

Spinal muscular atrophy (SMA) causes the degeneration of motor neurons in the spinal cord. Treatments including nusinersen, risdiplam, and onasemnogene abeparvovec have been shown to be effective in reducing symptoms, with recent studies suggesting greater effectiveness when treatment is initiated in the presymptomatic stage. This systematic review synthesises findings from prospective studies of presymptomatic treatment for 5q SMA published up to December 2023. The review identified three single-arm interventional studies of presymptomatic treatment (NURTURE, RAINBOWFISH, and SPR1NT), six observational studies comparing presymptomatic or screened cohorts versus symptomatic cohorts, and twelve follow-up studies of screened cohorts only (i.e., babies identified via newborn screening for SMA). Babies with three *SMN2* copies met most motor milestones in the NURTURE study of nusinersen and in the SPR1NT study of onasemnogene abeparvovec. Babies with two *SMN2* copies in these two studies met most motor milestones but with some delays, and some required ventilatory or feeding support. The RAINBOWFISH study of risdiplam is ongoing. Naïve comparisons of presymptomatic treatment in SPR1NT, versus untreated or symptomatic treatment cohorts, suggested improved outcomes in patients treated presymptomatically. Comparative observational studies supported the finding that presymptomatic treatment, and early treatment following screening, may improve outcomes compared with treatment at the symptomatic stage. Further research should assess the long-term clinical outcomes and cost-effectiveness of presymptomatic treatment for SMA.

## 1. Introduction

Spinal muscular atrophy (SMA) is an autosomal recessive disease associated with the degeneration of lower motor neurons in the anterior horn of the spinal cord and brainstem. SMA can lead to symmetrical muscle weakness, atrophy, and paralysis in late-stage severe disease. The onset of neuromuscular weakness ranges from birth to adulthood. Historically, SMA was classified into discrete types based on the age of onset of weakness, with SMA type 0 presenting neonatally and type 4 in early adulthood. It is now apparent that SMA spans a spectrum of disease, without discrete subtypes. The vast majority of cases of SMA (95%) are due to a homozygous deletion of exon 7 and 8 of the survival motor neuron 1 (*SMN1*) gene [1]. A minority are compound heterozygotes, where one copy of *SMN1* is deleted and the other has a missense variant. Overall, these genetic changes lead to a decrease in the functional survival motor neuron (SMN) protein and ultimately lead to patients developing SMA. The related survival motor neuron 2 (*SMN2*) gene can also make SMN protein, but only around 10% of the SMN protein from the *SMN2* gene is functional. Therefore, *SMN2* can partially compensate for deletions or pathogenic variants in *SMN1*. People can have multiple copies of the *SMN2* gene, with a higher number of *SMN2* copies generally correlating with reduced disease severity [2].

Treatments for SMA include nusinersen (Spinraza) [3], which is an antisense oligonucleotide designed to modify the product of the *SMN2* gene to produce more functional SMN protein; risdiplam (Evrysdi), which is a small molecule drug that targets the *SMN2* gene to produce more SMN protein [4]; and onasemnogene abeparvovec (Zolgensma), which is a gene therapy which expresses the SMN protein [5]. A summary of the regulatory and reimbursement status of the three drugs is provided in Table 1.

Recently, the treatment of SMA in the presymptomatic stage has been suggested to improve outcomes compared to the treatment of symptomatic disease. Presymptomatic treatment may be facilitated by identifying babies at an early stage via newborn screening [6,7]. It is important to further understand the impact of earlier diagnosis and treatment on patient outcomes.

We report a systematic review of prospective studies of pharmacological treatments for the presymptomatic treatment of SMA, to assess the impact on motor milestones and other outcomes.

## 2. Review Methods

### 2.1. Aims of Review

This systematic review aimed to summarise prospective studies assessing the impact of pharmacological treatments for the presymptomatic treatment of SMA on patient outcomes including survival; motor milestones; ventilation and feeding requirements; quality of life; and adverse events.

### 2.2. Search Strategy

Searches of MEDLINE, Embase, and the Cochrane Library were conducted to cover the period from January 2018 to December 2023; earlier publications were sought from a previous review conducted on behalf of the UK National Screening Committee [8]. Thesaurus and free-text terms for SMA (plus synonyms) were combined with ISSG search filters to identify randomised controlled trials (RCTs) and observational studies. The search strategy is provided in Appendix A. Recent reviews and relevant studies were also checked, and experts were consulted to identify any additional studies.

### 2.3. Inclusion Criteria

The review included prospective studies of pharmacological treatments for the presymptomatic treatment of 5q SMA. Studies comparing a presymptomatic or screened cohort versus a symptomatic cohort were eligible for inclusion, as were studies following up screened cohorts only. “Screened cohorts” consisted of babies identified via newborn screening for SMA, and generally included patients with and without symptoms at treatment initiation. Studies restricted to symptomatic patients were not included. All pharmacological interventions for the presymptomatic treatment of SMA were eligible for inclusion. Studies could include any comparator or no comparator. Relevant outcomes included survival; motor milestones (sitting; standing with assistance; standing independently; crawling; walking with assistance; walking independently); ventilation and feeding requirements; quality of life; and adverse events. All prospective study types were eligible for inclusion, including RCTs, single-arm interventional studies, and prospective real-world (observational) studies, including follow-ups of patients identified via newborn screening. Retrospective studies were not included.

### 2.4. Study Selection and Data Extraction

References were checked for inclusion by one reviewer and a 10% sample was checked by a second reviewer early in the process to check for consistency in inclusion decisions. Data for all studies were extracted by one reviewer and checked by another. Data were extracted relating to the study type, setting, population, intervention, comparator (if any), follow-up duration, and outcomes as listed above.

### 2.5. Risk of Bias Assessment

The risk of bias within the included studies was assessed using items from the Newcastle–Ottawa Scale [9], which were applicable to single-arm studies.

### 2.6. Approach to Synthesis

Evidence was presented via narrative synthesis and tabulation. Studies were tabulated according to the study type and intervention type. Outcome data were sub-grouped by *SMN2* copy number, due to its major impact on symptom severity.

## 3. Results

### 3.1. Volume and Type of Included Studies

**Figure 1:**
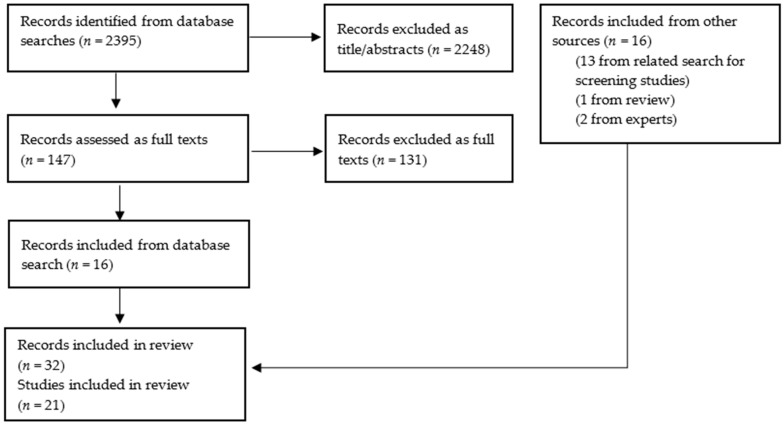
PRISMA flow chart.

The search identified 2395 references from the database search and 16 from other sources. The review included 21 studies in total, within 32 references. The review identified three single-arm interventional studies of presymptomatic treatment: the NURTURE study of nusinersen [10,11,12]; the RAINBOWFISH study of risdiplam [13,14]; and the SPR1NT study of onasemnogene abeparvovec [7,15,16,17]. In addition, the review identified six prospective observational studies comparing a presymptomatic (or screened) cohort versus a symptomatic cohort [18,19,20,21,22,23,24], as well as twelve studies reporting prospective follow-ups of screened cohorts only [25,26,27,28,29,30,31,32,33,34,35,36,37,38,39]. No RCTs of presymptomatic treatment were identified. A PRISMA flow diagram is shown in Figure 1.

### 3.2. Interventional Single-Arm Studies

The review identified three single-arm interventional studies of presymptomatic treatment as follows. The NURTURE study of nusinersen is a phase 2, multicentre, open-label, single-arm study which enrolled 25 babies with presymptomatic SMA, i.e., no clinical signs or symptoms of SMA (15 with two *SMN2* copies and 10 with three *SMN2* copies) [10,11]. Babies started to receive nusinersen via intrathecal injection at age ≤ 6 weeks and received four loading doses followed by a maintenance dose every 4 months. The median age at first dose was 19 days (range 8–41) for babies with two *SMN2* copies and 23 days (range 3–42) for babies with three *SMN2* copies. Patients have so far been treated for 5 years and will be followed for 8 years; however, the intended duration of treatment with nusinersen has not been clearly reported.

The SPR1NT study of onasemnogene abeparvovec (gene therapy) is a phase 3, multicentre, open-label, single-arm study which enrolled 29 babies with presymptomatic SMA, i.e., no clinical evidence of neuromuscular disease (14 with two *SMN2* copies and 15 with three *SMN2* copies) [7,15]. Babies received onasemnogene abeparvovec as a one-off intravenous infusion at age ≤ 6 weeks. The median age of infusion was 21 days (range 8–34) for babies with two *SMN2* copies and 32 days (range 9–43) for babies with three *SMN2* copies. The planned follow-up duration was 18 months for the two-copy cohort and 2 years for the three-copy cohort.

The RAINBOWFISH study of risdiplam is a multicentre, open-label, single-arm study [13,14]. Babies with presymptomatic SMA will receive risdiplam orally once daily, starting at age ≤ 6 weeks. The planned treatment duration is at least 5 years (2 years main study plus 3 years open-label extension). The study was still ongoing as of May 2024. As of 22 February 2022, 26 babies had been enrolled and recruitment was complete. A preliminary analysis of seven patients receiving risdiplam for at least 12 months is available as a conference abstract/poster.

Motor milestones from the three single-arm interventional studies are shown in Table 2, while Table 3 presents respiratory and swallowing outcomes as well as additional motor and neurological summary data. The tables are sub-grouped by *SMN2* copy number (two or three copies). For the motor milestones in Table 2 (sitting, standing and walking), the NURTURE study used WHO criteria and WHO developmental windows; the SPR1NT study used BSID-III criteria and WHO developmental windows; and the RAINBOWFISH study used BSID-III or HINE-2 criteria and WHO developmental windows.

#### 3.2.1. Outcomes in Babies with Two *SMN2* Copies

In the 5-year follow up of the NURTURE study of nusinersen in presymptomatic SMA (two *SMN2* copy cohort) [10,11,12], all 15 babies remained alive, all sat independently (11/15 within the normal developmental window), 14/15 crawled (6/15 in normal window), all stood with assistance (9/15 in normal window), 13/15 stood independently (4/15 in normal window), 14/15 walked with assistance (6/15 in normal window) and 13/15 walked independently (6/15 in normal window). No babies required tracheostomy or permanent ventilation, but 4/15 had respiratory support initiated during acute illnesses. In terms of feeding, 5/15 required a gastrostomy tube due to dysphagia (*n* = 3) or low weight (*n* = 2), but all continued to grow and gain weight.

In the SPR1NT study of onasemnogene abeparvovec in presymptomatic SMA (two *SMN2* copy cohort with 18-month follow-up) [7,16,17], all 14 babies remained alive, all sat independently (11/14 within normal window), 9/14 crawled (4/14 in normal window), all stood with assistance (6/14 in normal window), 11/14 stood independently (7/14 in normal window), 11/14 walked with assistance (6/14 in normal window) and 9/14 walked independently (5/14 in normal window). None required permanent ventilation, mechanical respiratory support, or nutritional support, and 13/14 maintained body weight through 18 months.

The SPR1NT study also reports a naïve comparison versus a matched cohort of untreated SMA type 1 patients with two copies of *SMN2* (*n* = 23), as well as versus studies of onasemnogene abeparvovec in symptomatic SMA type 1. In the untreated cohort, no babies achieved independent sitting, standing, or walking, whilst in the symptomatic trials, the proportions achieving these milestones appeared substantially lower than in the SPR1NT study (Table 2). The proportion surviving without permanent ventilation and the proportion maintaining body weight appeared higher in SPR1NT than in the untreated cohort and the symptomatic studies (Table 3). However, there was little detail on precise comparability between the various cohorts in terms of other factors potentially impacting severity.

In the preliminary analysis of the RAINBOWFISH study of risdiplam [13] in presymptomatic SMA (two *SMN2* copy cohort), all four babies were alive at a follow-up of at least 12 months, and all sat independently (two of four within normal window). For standing and walking milestones, two of four babies remained within the developmental window so they may have been yet to achieve the milestones, while of the other two babies, both stood independently, both crawled, and one walked independently. None required permanent ventilation and all maintained swallowing and feeding abilities. However, it was unclear whether there was some selection bias when restricting the analysis to babies receiving risdiplam for at least 12 months.

#### 3.2.2. Outcomes in Babies with Three *SMN2* Copies

Babies with three copies of *SMN2* generally had better outcomes than the two-copy cohorts (Table 2). In the 5-year follow up of the NURTURE study (three *SMN2* copy cohort) [10,11,12], all 10 babies remained alive, and all sat independently, crawled, stood independently, and walked independently, within the normal developmental windows. No babies required tracheostomy, permanent ventilation, or respiratory support. None required a gastrostomy tube, and all continued to grow and gain weight (Table 3).

In the SPR1NT study (three *SMN2* copy cohort with two-year follow-up) [15,16], all 15 babies remained alive, 14/15 sat independently (11/15 within normal window), 14/15 crawled (13/15 in normal window), 14/15 stood with assistance (11/15 in normal window), all stood independently (14/15 in normal window), 14/15 walked with assistance (13/15 in normal window) and 14/15 walked independently (11/15 in normal window). The fifteenth baby was noted to have walked independently but this was not captured on video. None required permanent ventilation, mechanical respiratory support, or a feeding tube, while 10/15 maintained body weight without feeding support through 24 months, and all achieved the body weight target by the end of the study.

The SPR1NT study also reports a naïve comparison versus a matched untreated cohort of patients with any SMA type and three copies of *SMN2* (*n* = 81). In the untreated cohort, 24% achieved independent standing and 21% achieved independent walking, which was substantially lower than SPR1NT participants; other motor milestones and ventilation and feeding outcomes were not reported, and there was little detail on exactly how comparable these various cohorts were.

In the preliminary analysis of the RAINBOWFISH study of risdiplam [13] (three or more *SMN2* copy cohort), all three babies were alive at a follow-up of at least 12 months, and all sat independently (one of three within normal window). In addition, all three stood independently, crawled, and walked independently, all within the normal windows. None required permanent ventilation and all maintained swallowing and feeding abilities.

### 3.3. Comparative Observational Studies

The review identified six prospective observational studies comparing a presymptomatic (or screened) cohort versus a symptomatic cohort (Table 4 and Table 5) [18,19,20,21,22,23,24]. Three studies used European registry data: one using the SMArtCARE registry across 70 centres in Germany, Austria, and Switzerland [22]; another using SMArtCARE across 18 centres in Germany and Austria [23]; and one using the Swiss Registry for Neuromuscular Disorders [21]. A further study used the RESTORE registry in 7 countries, mainly USA [24]. One study was conducted at a single centre in Belgium [20], and another at a single centre in Australia [18,19]. Three of the studies focussed only on patients receiving onasemnogene abeparvovec [21,23,24].

Three studies grouped patients as presymptomatic versus symptomatic [20,21,23], whereas three studies grouped patients as identified via screening versus identified via symptoms [18,22,24]; in the latter case, the screened group generally included some patients who had symptoms at treatment, but were generally still treated earlier than those identified via symptoms.

#### 3.3.1. Two *SMN2* Copies

Among patients with two *SMN2* copies, the only study with comparative data was the study by Kariyawasam et al. (2023) [18,19], in which patients identified via screening were treated earlier (median age 1 month) than patients identified via symptoms (median age 12 months) (Table 4). More treated patients in the screening group than in the symptoms group achieved standing (8/8 versus 1/7) and walking (5/8 versus 0/7), despite the fact that 4 of 8 treated patients in the screening group had some symptoms at the time of treatment.

#### 3.3.2. Three *SMN2* Copies

Among patients with three *SMN2* copies, in the study by Ngawa et al. (2023) [20], patients were treated earlier in the presymptomatic group (most within 1 month) than in the symptomatic group (10–16 months), and more patients in the presymptomatic cohort than in the symptomatic cohort achieved standing (4/5 versus 0/3) and walking (3/5 versus 0/3) (Table 4). In Kariyawasam et al. [18,19], patients with three *SMN2* copies identified via screening were treated earlier (median age 1 month) than patients identified via symptoms (median age 12 months), and more patients in the screening group than in the symptoms group achieved standing (5/5 versus 3/8) and walking (5/5 versus 1/8). Stettner et al. (2023) [21] also reported on patients with three *SMN2* copies but numbers were too small to make a robust comparison.

#### 3.3.3. Four or More *SMN2* Copies

The only study with comparative data on patients with four or more *SMN2* copies was the study by Kariyawasam et al. (2023) [18,19], but numbers were too small to make a robust comparison (Table 4).

#### 3.3.4. Mixed *SMN2* Copy Number Cohorts

Three studies reported on mixed cohorts in terms of *SMN2* copies (Table 4). Schwartz et al. (2024) [22] compared 44 screened patients (11 with symptoms at treatment; 70% with two copies and 30% with three copies of *SMN2*) versus 190 patients identified via symptoms (58% with two copies and 42% three copies of *SMN2*). More patients in the screening group than in the symptoms group achieved sitting (91% versus 74%) and independent walking (64% versus 15%). Weiss et al. (2022) [23] present data on patients with two or three *SMN2* copies, but little data are presented on the comparison between presymptomatic and symptomatic groups. Similarly, in the study by Servais et al. (2024) [24], which included patients with 1–4 *SMN2* copies, more patients in the screening group than in the symptoms group achieved independent walking (16/32 versus none).

#### 3.3.5. Respiratory and Feeding Outcomes

Two studies compared requirements for respiratory and feeding support in cohorts that were mixed in terms of *SMN2* copy number (Table 5). Both Schwartz et al. (2024) [22] and Kariyawasam et al. (2023) [18,19] reported more babies requiring ventilatory and feeding support in the symptomatic groups than in the screened groups, both at baseline and post-treatment.

### 3.4. Prospective Follow-Up of Screened Cohorts

The review also identified 12 studies reporting prospective follow-ups of screened cohorts only, without a symptomatic comparator cohort [25,26,27,28,29,30,31,32,33,34,35,36,37,38,39] (Table 6). These included cohorts from newborn screening studies in Belgium [27]; Germany [28,29,30,31]; Norway [32]; USA (California [34], Georgia [25], Massachusetts [33], New York State [26], North Carolina [35]); the RESTORE registry (seven countries, mainly USA) [39]; Japan (Kumamoto) [38]; Japan (Hyogo) [36]; and Taiwan [37].

These studies mainly reported on small numbers of patients with differing numbers of *SMN2* copies, treated presymptomatically or at an early symptomatic stage, with different treatments, and with varying durations of follow-up and varying completeness of outcome reporting. Almost all patients were identified via newborn screening, with a minority identified via other methods (symptoms or family history). Follow-up varied from a few months to one or two years. It is possible that there may have been overlap with patients in the three interventional studies described earlier. These data are presented in Table 6, sub-grouped according to *SMN2* copy number.

#### 3.4.1. One *SMN2* Copy

Two studies reported on a total of three babies with one *SMN2* copy; two were not treated and later died, while one received risdiplam but had severe symptoms at follow-up [25,26] (Table 6).

#### 3.4.2. Two *SMN2* Copies

Ten studies reported on a total of 73 babies with two *SMN2* copies [25,26,27,28,30,31,32,33,34,35,36,37] (Table 6). Around a third were presymptomatic and around two thirds were early symptomatic (where this was reported). All except seven babies received treatment, with most receiving nusinersen and/or onasemnogene abeparvovec, three receiving risdiplam, and some receiving more than one treatment. The majority of babies were treated at around 1 month of age (with age at treatment ranging from 0.4 to 6 months). Most babies were alive at follow-up, though the seven untreated babies died (some were untreated due to comorbidities). Of the babies receiving treatment, some met motor milestones while others were delayed. The majority of treated patients did not require respiratory or feeding support, but a minority required these interventions.

#### 3.4.3. Three *SMN2* Copies

Eight studies reported on a total of 46 babies with three *SMN2* copies [25,26,27,28,32,34,37,38] (Table 6). The majority were presymptomatic and a few were early symptomatic. All except five babies received treatment, all with nusinersen, onasemnogene abeparvovec, or both. Again, the majority of babies were treated at around 1 month of age (with age at treatment ranging from 0.5 to 6 months). All those with follow-up data were alive at follow-up. Most were asymptomatic at follow-up and met motor milestones, though a few had motor delays. No treated patients required respiratory or feeding support, though this was not well-reported.

#### 3.4.4. Four or More *SMN2* Copies

Nine studies reported on a total of 51 babies with four or more *SMN2* copies [25,26,27,28,29,32,33,34,37,39] (Table 6). The majority were presymptomatic (where reported). Around a third were untreated, and the remainder received mainly nusinersen and/or onasemnogene abeparvovec, while one received risdiplam. Age of treatment ranged from 1 to 36 months. All were alive at follow-up. Most treated patients were asymptomatic at follow-up and met motor milestones. Of the untreated patients, some were asymptomatic at follow-up while others had symptoms. No patients required respiratory or feeding support, though this was not well-reported.

### 3.5. Risk of Bias in the Included Studies

The risk of bias in the included studies is shown in Table 7. In terms of the three interventional single-arm studies, the representativeness of the patient cohort to a screened population was judged to be unclear; this was because patients were required to be presymptomatic at the start of treatment, whereas the cohorts of screened patients are likely to include both presymptomatic and early symptomatic patients. Fidelity to the intended intervention was judged to be positive. The outcome assessment was not blinded, which could have led to bias. All three studies had at least a 1-year follow-up, although in practice it will be important to follow patients for several years to assess whether motor milestones are maintained. NURTURE and SPR1NT analysed all enrolled patients, while the preliminary analysis of RAINBOWFISH only included seven patients with treatment of at least 1 year, which may have led to selection bias if some patients had discontinued before 1 year, though this is not clear. In addition, all three studies involved small patient numbers per sub-group for *SMN2* copy number, and no studies had a randomised controlled design; this is likely due to the low prevalence and severity of the condition, but this means that it is difficult to robustly compare outcomes between treatments, or versus no treatment or symptomatic treatment.

In terms of the prospective observational studies and screening follow-up studies, all were assumed to involve a representative patient cohort since most followed up all positive cases from screening. Fidelity to the intended intervention was judged to be unclear as little detail of treatment was reported. No studies reported blinded outcome assessment. Follow-up duration was very variable, both between studies and between patients within a study, making it difficult to assess motor milestones. Some studies analysed all included patients, while some only reported data for a subset of patients. In addition, observational studies each reported on small numbers of patients with differing *SMN2* copy numbers, symptom profiles, treatment types, follow-up durations, and outcome reporting, making the results difficult to interpret.

### 3.6. Quality of Life

For the NURTURE study of nusinersen, caregiver quality of life was reported in a conference abstract [12], in which caregivers of patients were assessed using the Assessment of Caregiver Experience with Neuromuscular Disease (ACEND) and the Pediatric Quality of Life Inventory (PedsQL) Generic Core Scale (GCS) and Neuromuscular Module (NM). Mean scores on ACEND and PedsQL were generally higher among caregivers of participants with three versus two *SMN2* copies. There was an overall pattern of increases in ACEND mean scores among caregivers between first and final assessment in physical impact subdomains: feeding/grooming/dressing, transfer, and mobility (for both the two-copy and three-copy cohorts). Near-maximum ACEND mean scores were maintained for the sitting/play physical impact subdomain, regardless of *SMN2* copy number.

In addition, a follow-up of the Belgian screening study reported quality of life for a cohort of patients “not identified via symptoms” (identified via screening or sibling; presymptomatic or early symptomatic; *n* = 14) versus two cohorts of symptomatic patients (treated, *n* = 42; and untreated, *n* = 93) [40]. Patients “not identified via symptoms” received either nusinersen, onasemnogene abeparvovec, or risdiplam. Health-related quality of life and utility scores were generally higher in patients “not identified by symptoms” than in symptomatic patients, though the analysed numbers were small. On the Health Utilities Index (HUI), *N* = 3 analysed patients in the “not identified by symptoms” group (two or three *SMN2* copies) received a maximum or close to maximum score. On the PedsQL Family Impact scale, *N* = 13 analysed patients in the “not identified by symptoms” group (two, three, or four *SMN2* copies) were “similarly impacted” as symptomatic patients. On the Peds QL GCS and NM subscales, *N* = 4 analysed patients in the “not identified by symptoms” group (two or three *SMN2* copies) had higher scores than symptomatic patients, but the sample size was too small for formal comparison. On the EQ-5D, only *N* = 1 patient was analysed in the “not identified by symptoms” group.

### 3.7. Adverse Events

Adverse events (AEs) are summarised in Table 8. AE data are taken from the study reports and information on special warnings and precautions from the regulatory summaries from the European Medicines Agency (EMA) and the US Food and Drugs Administration (FDA).

For nusinersen, special warnings and precautions (from the EMA and FDA) [41,42] included the following: risk of adverse effects due to lumbar puncture; risk of thrombocytopenia, coagulation abnormalities, and renal toxicity (as observed with other antisense oligonucleotides); and hydrocephalus. In the NURTURE study [10], serious AEs occurred in 12/25 (48%) patients, but none were considered treatment-related by the investigators. AEs potentially related to lumbar puncture occurred in 13/25 (52%) patients. All AEs resolved despite continued treatment, except for proteinuria (*n* = 1) and clonus (*n* = 1). Potentially treatment-related AEs in the NURTURE study included increases in aminotransferases or alkaline phosphatase (2/25), protein in the urine (3/25), pyrexia (1/25), allergic dermatitis (1/25), headache (1/25), and rash (1/25). A list of all AEs is provided in Table 8.

For risdiplam, special warnings and precautions [43,44] included the following: embryo–foetal toxicity and effects on male fertility (both based on animal studies); and retinal toxicity (from non-clinical safety studies). In the RAINBOWFISH study [13], no serious AEs were reported in the 18 patients analysed in a conference presentation, though 8/26 (31%) patients reported on ClinicalTrials.gov [14] for the RAINBOWFISH study had serious AEs (though not necessarily treatment-related); these included urinary tract infection (2/26); gastroenteritis (2/26); constipation (1/26); femur fracture (1/26); soft tissue injury (1/26); and neonatal jaundice (1/26). In addition, 2/18 (11%) reported treatment-related AEs of any grade (1/18 diarrhoea; 1/18 skin discoloration) [13]. A list of all AEs is provided in Table 8.

For onasemnogene abeparvovec, special warnings and precautions [45,46] included the following: acute liver failure with fatal outcomes, acute serious liver injury and elevated aminotransferases (corticosteroids are recommended for all patients to prevent this); systemic immune responses; transient thrombocytopenia; thrombotic microangiopathy; cardiac effects and increased cardiac troponin-I levels; and theoretical risk of tumorigenicity due to the integration of the viral vector into the genome. In the SPR1NT study [7,15], serious AEs occurred in 5/14 patients (36%) in the two-copy cohort and 3/15 (20%) in the three-copy cohort but none were considered treatment-related by the investigators. All patients received oral prednisolone to attenuate the inflammatory response. AEs of special interest occurred as follows: hepatotoxicity (7/29); thrombocytopenia (5/29); cardiac AEs (5/29); thrombotic microangiopathy (2/29); and sensory abnormalities suggestive of dorsal root ganglionopathy (4/29). Lists of all AEs and treatment-related AEs are provided in Table 8. In addition, two cohort studies reported on AEs for onasemnogene abeparvovec, one based on the RESTORE registry [24] (*n* = 97 screened patients and *n* = 70 symptomatic patients) and one study in Switzerland [21] (*n* = 9 presymptomatic patients); these are summarised in Table 8.

## 4. Discussion

Three single-arm interventional studies assessed three different presymptomatic SMA treatments (nusinersen, onasemnogene abeparvovec, and risdiplam). In the NURTURE study of nusinersen and the SPR1NT study of onasemnogene abeparvovec, babies with three *SMN2* copies met most motor milestones, while babies with two *SMN2* copies met most motor milestones but with some delays, and some required ventilatory or feeding support. The RAINBOWFISH study of risdiplam is ongoing. Naïve comparisons of the SPR1NT cohort versus an untreated cohort, and versus studies of symptomatic treatment, suggested improved outcomes in patients treated presymptomatically.

This review also identified six comparative observational studies, three comparing presymptomatic versus symptomatic cohorts, and three comparing a screened cohort (including a mix of presymptomatic and early symptomatic patients) against a cohort identified via symptoms. These studies suggested that patients in the screening (or presymptomatic) cohorts received earlier treatment and may have improved outcomes compared with patients identified via symptoms, both for patients with two and three *SMN2* copies. An additional 12 screening follow-up studies (without a comparator group) supported the finding that patients receiving early treatment often met motor milestones; those with three *SMN2* copies had better outcomes than those with two copies.

Another recent review summarised sitting and walking outcomes across studies for SMA patients identified via newborn screening, and drew similar conclusions to our review [47]. Additional reviews [48,49,50,51,52,53,54] focussed mainly on summarising the three interventional studies, while a review of onasemnogene abeparvovec [55] covered mainly symptomatic studies but also identified a presymptomatic study. In summary, current data indicate that the presymptomatic (or early symptomatic) treatment of SMA appears to improve motor and functional outcomes to a greater extent than treatment at the symptomatic stage, at least in the short-term. This suggests that the identification of babies with SMA via newborn screening may be valuable in enabling earlier treatment.

The results from studies of presymptomatic treatment appear positive so far, though there were some limitations. So far, the median follow-up is 5 years in the NURTURE study and 18 months (two *SMN2* copy cohort) or 2 years (three copy cohort) in the SPR1NT study. Further follow-up is needed to determine long-term outcomes for these children. In addition, patients identified via newborn screening sometimes displayed early symptoms of SMA, but symptomatic patients were excluded from the three interventional studies. Furthermore, the NURTURE and SPR1NT studies only assessed patients with two or three copies of *SMN2*, while there is greater uncertainty regarding the management of patients with four *SMN2* copies, who may have milder late-onset disease. There are also limited data on babies with one *SMN2* copy, who are likely to be severely affected at birth. The cost-effectiveness of strategies involving newborn screening and presymptomatic treatment is another area of current research. Further data would be valuable regarding adverse effects of the different treatments in larger cohorts, as well as the impact of the treatments on quality of life for both patients and carers. In addition, the further evaluation of combinations or sequences of disease-modifying therapies will be an important area for future study [56,57].

All three treatments (nusinersen, onasemnogene abeparvovec, and risdiplam) have been recommended by the National Institute for Health and Care Excellence (NICE) in England and Wales for the presymptomatic treatment of 5q SMA. The nusinersen and risdiplam recommendations are currently under review following additional data collection, while gene therapy provision is subject to a commercial arrangement. In addition, risdiplam is restricted to patients with 1–4 *SMN2* copies, while onasemnogene abeparvovec is restricted to patients with up to 3 *SMN2* copies.

Ongoing research worldwide is assessing the long-term clinical outcomes, as well as the cost-effectiveness, of newborn screening and presymptomatic treatment of 5q SMA.

## 5. Conclusions

Presymptomatic treatment, and early treatment following screening, may improve outcomes in babies with SMA compared with treatment at the symptomatic stage, based on single-arm interventional studies and comparative observational studies. Ongoing evaluations will assess the long-term clinical and cost-effectiveness of the presymptomatic treatment of 5q SMA.

## Figures and Tables

**Table 1:** Regulatory and reimbursement status.

	Nusinersen	Risdiplam	Onasemnogene Abeparvovec
Brand name	Spinraza	Evrysdi	Zolgensma
Manufacturer	Biogen Idec	Roche	Novartis Gene Therapies
Mechanism of action	Antisense oligonucleotide designed to modify the product of the *SMN2* gene to produce more functional SMN protein	Small molecule *SMN2* splicing modifier which targets the *SMN2* gene to produce more SMN protein	Gene therapy product within a recombinant viral vector, which expresses human SMN protein
Mode of delivery	Multiple intrathecal injections (four loading doses followed by maintenance dose every 4 months)	Oral administration on a daily basis	Administered once as a single-dose intravenous infusion
Marketing authorisation (EMA)	Treatment of 5q SMA (2017; updated 2022)	Treatment of 5q SMA in patients with a clinical diagnosis of SMA type 1, type 2, or type 3, or with one to four *SMN2* copies (2021)	Treatment of 5q SMA with a bi-allelic mutation in the *SMN1* gene and either a clinical diagnosis of SMA type 1, or up to 3 copies of the *SMN2* gene (2020; updated 2022)
Marketing authorisation (FDA)	Treatment of SMA in paediatric and adult patients (2016)	Treatment of SMA in patients 2 months of age and older (2020)	Treatment of paediatric patients less than 2 years of age with SMA with bi-allelic mutations in the *SMN1* gene (2019; updated 2023)
NICE recommendation (symptomatic SMA)	Recommended in 2019 for treatment of 5q SMA types 1, 2, or 3 subject to a managed access agreement and further data collection (TA588). Guidance under review (expected Dec 2024)	Recommended in 2021 for treatment of 5q SMA types 1, 2, or 3, subject to a managed access agreement and further data collection (TA755). Guidance under review (expected Dec 2024)	Recommended in 2021 for treatment of 5q SMA type 1 with a bi-allelic *SMN1* mutation in babies aged 6 months or younger (or 7 to 12 months if agreed by national multidisciplinary team), if not requiring tracheostomy or permanent ventilation for >16 h/day, and subject to a commercial arrangement (HST15)
SMC recommendation (symptomatic SMA)	Recommended in 2018 for treatment of symptomatic type 1 5q SMA, and in 2019 for treatment of types 2 and 3 SMA (for up to 3 years while further evidence generated)	Recommended in 2022 for treatment of 5q SMA in patients aged 2 months and older with a clinical diagnosis of SMA types 1, 2, or 3	Recommended in 2021 for treatment of 5q SMA with a bi-allelic mutation in the *SMN1* gene and a clinical diagnosis of SMA type 1
NICE recommendation (presymptomatic SMA)	Recommended in 2019 for treatment of presymptomatic 5q SMA, subject to a managed access agreement and further data collection (TA588). Guidance under review (expected Dec 2024)	Recommended in 2021 for treatment of presymptomatic 5q SMA and 1 to 4 *SMN2* copies, subject to a managed access agreement and further data collection (TA755). Guidance under review (expected Dec 2024)	Recommended in 2023 for treatment of presymptomatic 5q SMA with a bi-allelic *SMN1* mutation and up to 3 copies of the *SMN2* gene in babies aged 12 months and under, subject to a commercial arrangement (HST24)
SMC recommendation (presymptomatic SMA)	No specific recommendations for presymptomatic SMA or patients with specific *SMN2* copy numbers	Recommended in 2022 for treatment of 5q SMA in patients aged 2 months and older with 1 to 4 *SMN2* copies	Recommended in 2021 for treatment of presymptomatic 5q SMA with a bi-allelic mutation in the *SMN1* gene and up to 3 copies of the *SMN2* gene (expected to develop SMA type 1)

Abbreviations: EMA, European Medicines Agency; FDA, Food and Drug Administration; NICE, National Institute for Health and Care Excellence; SMA, spinal muscular atrophy; SMC, Scottish Medicines Consortium; SMN, survival motor neuron.

**Table 2:** Single-arm interventional studies: Motor milestones.

Study	Study Type Setting	Population	Interventions Follow-Up	Survival	Sitting Independently ^a^	Crawling ^a^	Standing with Assistance ^a^	Standing Independently ^a^	Walking with Assistance ^a^	Walking Independently ^a^
**Two *SMN2* copies**										
NURTURE Crawford 2023 [10] De Vivo 2019 [11]	- Ph2, OL, MC, single-arm trial - 15 sites in 7 countries	Two *SMN2* copies (*n* = 15) - Babies (≤6 weeks) with presympt SMA - NR how diagnosed	- Nusinersen (intrathecally every 4 mo) - Median age at first dose: 19 days (range 8–41) - Median FU 4.9 yr (data Feb 2021)	- 15/15 (100%) alive at FU	- 15/15 (100%) sat independently - 11/15 (73%) within normal window	- 14/15 (93%) crawled - 6/15 (40%) within normal window	- 15/15 (100%) stood with assistance - 9/15 (60%) within normal window	- 13/15 (87%) stood independently - 4/15 (27%) within normal window	- 14/15 (93%) walked with assistance - 6/15 (40%) within normal window	- 13/15 (87%) walked independently - 6/15 (40%) within normal window
SPR1NT Strauss 2022 [7] Chaplin 2023 [17]	- Ph3, OL, MC, single-arm trial - 16 sites in 6 countries	Two *SMN2* copies (*n* = 14) - Babies (≤6 weeks) with presympt SMA - 5 via prenatal screening, 9 via newborn screening	- Onasemnogene abeparvovec via one-off infusion - Median age at infusion: 21 days (range 8–34) - FU to age 18 mo Compared with: - Untreated matched PNCR cohort (*n* = 23) - Symptomatic SMA type 1 studies ^b^	- 14/14 (100%) alive at 14 mo	- 14/14 (100%) sat independently by 18 mo - 11/14 (79%) within normal window Untreated: - 0/23 (0%) (*p* < 0.0001) Symptomatic ^b^ - START 9/12 (75%) - STR1VE-US 14/22 (64%) - STR1VE-EU 14/32 (44%) ^c^	- 9/14 (64%) crawled - 4/14 (29%) within normal window Untreated: NR	- 14/14 (100%) stood with assistance - 6/14 (43%) within normal window Untreated: NR	- 11/14 (79%) stood independently by 18 mo - 7/14 (50%) within normal window Untreated: - 0/23 (0%) Symptomatic ^b^ - START 2/12 (17%) - STR1VE-US 1/22 (5%) - STR1VE-EU 1/33 (3%) ^c^	- 11/14 (79%) walked with assistance - 6/14 (43%) within normal window Untreated: NR	- 9/14 (64%) walked independently by 18 mo - 5/14 (36%) within normal window Untreated: - 0/23 (0%) Symptomatic SMA ^b^ - START 2/12 (17%) - STR1VE-US 1/22 (5%) - STR1VE-EU 1/33 (3%) ^c^
RAINBOW-FISH Finkel 2022 [13,14] (abst)	- OL, MC, single-arm trial - 7 sites in 7 countries	Two *SMN2* copies (*n* = 4) - Babies (≤6 weeks) with presympt SMA - NR how diagnosed	- Risdiplam orally once daily - Median age at first dose: NR - Analysed pts with ≥12 mo FU (data July 2021)	- 4/4 (100%) alive at FU	- 4/4 sat independently; 2/4 within normal window	- 2/4 crawled - 2/4 within normal window - Remaining 2/4 still within window (may achieve milestone)	NR	- 2/4 stood independently - 1/4 within normal window - Remaining 2/4 still within window (may achieve milestone)	NR	- 1/4 walked independently - 1/4 within normal window - Remaining 2/4 still within window (may achieve milestone)
**Three *SMN2* copies**										
NURTURE Crawford 2023 [10] De Vivo 2019 [11]	- Ph2, OL, MC, single-arm trial - 15 sites in 7 countries	Three *SMN2* copies (*n* = 10) - Babies (≤6 weeks) with presympt SMA - NR how diagnosed	- Nusinersen (intrathecally every 4 mo) - Median age at first dose: 23 days (range 3–42) - Median FU 4.9 yr (data Feb 2021)	- 10/10 (100%) alive at FU	- 10/10 (100%) sat independently - 10/10 (100%) within normal window	- 10/10 (100%) crawled - 10/10 (100%) within normal window	- 10/10 (100%) stood with assistance - 10/10 (100%) within normal window	- 10/10 (100%) stood independently - 10/10 (100%) within normal window	- 10/10 (100%) walked with assistance - 9/10 (90%) within normal window	- 10/10 (100%) walked independently - 10/10 (100%) within normal window
SPR1NT Strauss 2022 [15]	- Ph3, OL, MC, single-arm trial - 16 sites in 6 countries	Three *SMN2* copies (*n* = 15) - Babies (≤6 weeks) with presympt SMA - 13 via newborn screening; 1 via prenatal screening; 1 NR	- Onasemnogene abeparvovec via one-off infusion - Median age at infusion: 32 days (range 9–43) - FU to age 24 mo Compared with: - Untreated matched PNCR cohort (*n* = 81)	- 15/15 (100%) alive at 14 mo	- 14/15 (93%) sat independently by 24 mo - 11/15 (73%) within normal window Untreated cohort: NR	- 14/15 (93%) crawled - 13/15 (87%) within normal window Untreated: NR	- 14/15 (93%) stood with assistance - 11/15 (73%) within normal window Untreated: NR	- 15/15 (100%) stood independently by 24 mo - 14/15 (93%) within normal window Untreated: - 19/81 (24%) (*p* < 0.0001)	- 14/15 (93%) walked with assistance - 13/15 (87%) within normal window Untreated: NR	- 14/15 (93%) walked independently by 24 mo (1 additional not captured on video) - 11/15 (73%) within normal window Untreated: - 17/81 (21%) (*p* < 0.0001)
RAINBOW-FISH Finkel 2022 [13,14] (abst)	- OL, MC, single-arm trial - 7 sites in 7 countries	Three+ *SMN2* copies (*n* = 3) - Babies (≤6 weeks) with presympt SMA - NR how diagnosed	- Risdiplam orally once daily - Median age at first dose: NR - Analysed pts with ≥12 mo FU (data July 2021)	- 3/3 (100%) alive at FU	- 3/3 sat independently - 1/3 within normal window	- 3/3 crawled - 3/3 within normal window	NR	- 3/3 stood independently - 3/3 within normal window	NR	- 3/3 walked independently - 3/3 within normal window

^a^ Motor milestones: NURTURE used WHO criteria and WHO developmental windows; RAINBOWFISH used BSID-III or HINE-2 criteria [unclear which] and WHO developmental windows; SPR1NT used BSID-III criteria and WHO developmental windows. ^b^ START, STR1VE-US and STR1VE-EU are all single-arm studies of onasemnogene abeparvovec in infantile-onset symptomatic SMA type 1 with two *SMN2* copies. ^c^ STR1VE-EU includes denominators of *n* = 33 (all patients) or *n* = 32 (intention-to-treat) due to the exclusion of 1 patient from some of the analyses because of age of onset of treatment. Abbreviations: abst, abstract; FU, follow-up; MC, multicentre; mo, months; NR, not reported; OL, open-label; Ph, phase; PNCR, Pediatric Neuromuscular Clinical Research; presymp, presymptomatic; SMA, spinal muscular atrophy; *SMN2*, survival motor neuron 2; yr, years.

**Table 3:** Single-arm interventional studies: Respiratory and swallowing outcomes and motor scores.

Study	Population	Interventions, FU	Respiratory Outcomes	Feeding Outcomes	Other Motor and Neurological Outcomes
**Two *SMN2* copies**					
NURTURE Crawford 2023 [10]; De Vivo 2019 [11]; Kirschner 2022 [12] (abst)	Two *SMN2* copies (*n* = 15) - Babies with presympt SMA	- Nusinersen - Median FU 4.9 yr	- None required tracheostomy or permanent ventilation - 4/15 (27%) had respiratory support (≥6 h/day for ≥7 consecutive days), initiated during acute, reversible illnesses	- 5/15 (33%) required gastrostomy tube Reasons for tube placement: dysphagia (*n* = 3; 1 used as needed); low weight (*n* = 2) - 15/15 (100%) continued to grow and gain weight	- 12/15 (80%) achieved maximum CHOP INTEND score (score 64) - 10/15 at age 13 mo and 7/15 at age 24 mo had protocol-defined SMA symptoms. The 7 with symptoms all continued to grow, gain weight and achieve motor milestones
SPR1NT Strauss 2022 [7] Shell 2023 [16]	Two *SMN2* copies (*n* = 14) - Babies with presympt SMA	- Onasemnogene abeparvovec - FU to age 18 mo Compared with: - Untreated matched PNCR cohort (*n* = 23) - Symptomatic SMA type 1 studies ^a^	- 14/14 (100%) survived without permanent ventilation at 14 mo as per protocol - No mechanical respiratory support Untreated: - 6/23 (26%) survived without permanent ventilation (*p* < 0.0001) Symptomatic SMA ^a^ - STR1VE-US 20/22 (91%); - STR1VE-EU 31/32 (97%) survived without permanent ventilation ^b^	- None required nutritional support - 13/14 (93%) maintained body weight (≥3rd WHO percentile) through 18 mo - 14/14 (100%) swallowed normally, full oral nutrition, maintained pulmonary stability [Shell] Untreated: NR Symptomatic SMA ^a^ - STR1VE-US 14/22 (64%) maintained weight - STR1VE-EU 15/33 (65%) maintained weight ^b^	- CHOP INTEND scores increased rapidly during initial 3 mo after infusion, reached a median of 60 (range: 51–64) by 6 mo, and all (14/14, 100%) reached score of at least 58 by 18 mo
RAINBOWFISH Finkel 2022 [13] (abst)	Two *SMN2* copies (*n* = 4) - Babies with presympt SMA	- Risdiplam - Analysed pts with ≥12 mo FU	- 4/4 (100%) were alive without permanent ventilation at FU	- 4/4 (100%) maintained swallowing and feeding abilities and had not required hospitalisation	- Most achieved near-maximum CHOP INTEND score
**Three *SMN2* copies**					
NURTURE Crawford 2023 [10]; De Vivo 2019 [11]	Three *SMN2* copies (*n* = 10) - Babies with presympt SMA	- Nusinersen - Median FU 4.9 yr	- None required tracheostomy or permanent ventilation - None used respiratory support (≥6 h/day for ≥7 consecutive days)	- 0/10 (0%) required gastrostomy tube - 10/10 (100%) continued to grow and gain weight	- 10/10 (100%) achieved maximum CHOP INTEND score (score 64) - Mean CHOP INTEND scores increased steadily from baseline; stabilized around maximum score - Mean CHOP INTEND scores were higher in NURTURE than in ENDEAR (symptomatic SMA) - 2/10 at age 13 mo and 0/10 at age 24 mo had protocol-defined SMA symptoms
SPR1NT Strauss 2022 [15] Shell 2023 [16]	Three *SMN2* copies (*n* = 15) - Babies with presympt SMA	- Onasemnogene abeparvovec - FU to age 24 mo	- 15/15 (100%) survived without permanent ventilation at 14 mo - No mechanical respiratory support Untreated: NR	- None required feeding tube - 10/15 (67%) maintained body weight (≥3rd WHO percentile) without feeding support at all study visits through 24 mo - 15/15 (100%) reached ≥3rd WHO percentile for body weight by study end - 15/15 (100%) swallowed normally, full oral nutrition, maintained pulmonary stability [Shel] Untreated: NR	- [CHOP INTEND not reported]
RAINBOWFISH Finkel 2022 [13] (abst)	Three+ *SMN2* copies (*n* = 3) - Babies with presympt SMA	- Risdiplam - Analysed pts with ≥12 mo FU	- 3/3 (100%) were alive without permanent ventilation at FU	- 3/3 (100%) maintained swallowing and feeding abilities and had not required hospitalisation	- 3/3 (100%) achieved maximum CHOP INTEND score

^a^ STR1VE-US and STR1VE-EU are single-arm studies of onasemnogene abeparvovec in infantile-onset symptomatic SMA type 1 with two *SMN2* copies. ^b^ STR1VE-EU includes denominators of *n* = 33 (all patients) or *n* = 32 (intention-to-treat) due to the exclusion of 1 patient from some of the analyses because of age of onset of treatment. Abbreviations: abst, abstract; CHOP INTEND, Children’s Hospital of Philadelphia Infant Test of Neuromuscular Disorders; FU, follow-up; mo, months; NR, not reported; PNCR, Pediatric Neuromuscular Clinical Research; presympt, presymptomatic; SMA, spinal muscular atrophy; *SMN2*, survival motor neuron 2; yr, years.

**Table 4:** Comparative observational studies: Motor milestones.

Study	*SMN2* Copies	Presymptomatic and/or Screened Cohorts						Symptomatic Cohorts					
		Group	Interventions	Survival	Sitting	Standing	Walking	Group	Interventions	Survival	Sitting	Standing	Walking
Kariyawasam 2023 [18]; 2020 [19] Australia (1 centre) FU 24 mo	Two copies	Screened *N* = 9 (4 presympt, 5 sympt)	- 8 Nus or OA (median 1 mo) - 1 untreated (SMA+ comorbidities)	- 8/8 alive - 0/1 alive	- 8/8 sat - 0/1 sat	- 8/8 stood with asst; 7/8 stood alone - 0/1 stood	- 5/8 walked with asst; 3/8 walked alone - 0/1 walked	Sympt *N* = 9	- 7 Nus (median 12 mo) - 2 untreated (SMA + comorbidities)	- 7/7 alive - 0/2 alive	- 6/7 sat - 0/2 sat	- 1/7 stood with asst - 0/2 stood	- 0/7 walked - 0/2 walked
	Three copies	Screened *N* = 5 (4 presympt, 1 sympt)	- 5 Nus or OA (median 1 mo)	- 5/5 alive	- 5/5 sat	- 5/5 stood with asst; 5/5 stood alone	- 5/5 walked with asst; 5/5 walked alone	Sympt *N* = 8	- 8 Nus (median 12 mo)	- 8/8 alive	- 8/8 sat	- 3/8 stood with asst; 3/8 stood alone	- 1/8 walked with asst; 0/8 walked alone
	Four + copies	Screened *N* = 1 (presympt)	- 1 untreated	- 1/1 alive	- 1/1 sat	- 1/1 stood with asst; 1/1 stood alone	- 1/1 walked with asst; 1/1 walked alone	Sympt *N* = 1	- 1 Nu (median 12 mo)	- 1/1 alive	- 1/1 sat	- 1/1 stood with asst; 1/1 stood alone	- 0/1 walked with asst; 0/1 walked alone
Ngawa 2023 [20] Belgium (1 centre) FU 10–61 mo	Two copies							Sympt *N* = 8 Via scr/sympt Type 1	- 5 Nus + Ris (1–5 mo) - 3 OA (2–5 mo)	- 5/5 alive - 3/3 alive	- 4/5 sat - 3/3 sat	- 1/5 stood - 1/3 stood	- 0/5 walked - 0/3 walked
	Three copies	Presympt *N* = 5 Via scr/FH	- 1 Nus+Ris (1 mo) - 2 Nus (1–6 mo) - 1 OA (1 mo) - 1 Ris (1 mo)	- 1/1 alive - 2/2 alive - 1/1 alive - 1/1 alive	- 1/1 sat - 2/2 sat - 1/1 sat - 1/1 sat	- 1/1 stood - 2/2 stood - 1/1 stood - 0/1 stood	- 1/1 walked - 1/2 walked - 1/1 walked - 0/1 walked	Sympt *N* = 3 Via scr/sympt Type 1	- 2 Nus + Ris (10–12 mo) - 1 Nus (16 mo)	- 2/2 alive - 1/1 alive	- 1/2 sat - 1/1 sat	- 0/2 stood - 0/1 stood	- 0/2 walked - 0/1 walked
	Four copies	Presympt *N* = 2 Via scr/FH	- 2 Ris (1 mo)	- 2/2 alive	- 2/2 sat	- 1/2 stood	- 1/2 walked						
Stettner 2023 [21] Switzerland (registry) FU 6–20 mo	Two copies							Sympt *N* = 6 Type 1	- 4 OA (2–6 mo) - 2 Nus + OA (1–3 mo + 3–10 mo)	- 6/6 alive	- 3/6 sat	- 0/6 stood	- 0/6 walked
	Three copies	Presympt *N* = 2 Via FH	- 2 OA (1 mo)	- 2/2 alive	- 2/2 sat	- 2/2 stood indep	- 2/2 walked indep	Sympt *N* = 1 Type 2	- 1 OA (17 mo)	- 1/1 alive	- 1/1 sat	- 1/1 stood with asst	- 0/1 walked
Schwartz 2024 [22] Germany, Austria, Switzerland (SMArtCARE registry; 70 centres) FU ≥ 18 mo	Two/three copies	Screened *N* = 44 (33 presympt, 11 sympt) *SMN2*: - Two: 31 - Three: 13	- 12 Nus - 7 OA - 21 Nus + OA - 2 Nus + Ris - 2 untreated (mean 1 mo)	NR	- 40/44 (91%) sat	NR	- 28/44 (64%) walked indep, 18 (41%) in normal window; latter all presympt at start	Sympt *N* = 190 *SMN2*: One: 1 Two: 110 Three: 79	- 21 Nus - 66 OA - 4 Ris - 72 Nus + OA - 13 Nus + Ris - 5 untreated (mean 11 mo)	NR	- 141/190 (74%) sat	NR	- 28/190 (15%) walked indep, 11 (6%) in normal window
Weiss 2022 [23] Germany, Austria (SMArtCARE; 18 centres) FU 6 mo	Two/three copies	Presympt *N* = 6 NR how identified	- 6 OA (NR)	- 6/6 alive	NR	NR	NR	Sympt *N* = 50 Type 1 (*N* = 45) Type 2 (*N* = 5)	- 50 OA ± Nus (1–59 mo)	- 50/50 alive	NR	NR	NR
Servais 2024 [24] (RESTORE; 7 countries, mainly USA) FU 0–37 mo	1/2/3/4 copies	Screened *N* = 32 (presympt or sympt)	- 32 OA (0–72 mo)	NR	NR	- 16/32 stood indep (within window)	- 16/32 walked indep (10/32 within window); 20/32 walked with asst	Sympt *N* = NR	- OA (0–28 mo) (*N* = NR)	NR	NR	NR	- None walked indep

Abbreviations: asst, assistance; d, days; FU, follow-up; indep, independently; mo, months; NR, not reported; Nus, nusinersen; OA, onasemnogene abeparvovec; presympt, presymptomatic; Ris, risdiplam; SMA, spinal muscular atrophy; *SMN2*, survival motor neuron 2; sympt, symptomatic; wk, weeks; yr, years.

**Table 5:** Prospective comparative studies: Respiratory and swallowing outcomes and motor scores.

Study	*SMN2* Copies	Presymptomatic and/or Screened Cohorts					Symptomatic Cohorts				
		Group	Interventions	Other Motor Outcomes	Respiratory	Feeding	Group	Interventions	Other Motor Outcomes	Respiratory	Feeding
Kariyawasam 2023 [18]; 2020 [19] Australia FU 24 mo	Two/ three/ four copies	Screened *N* = 15 (9 presympt, 6 sympt)	- 13 Nus or OA (median 1 mo) - 2 untreated	NR	- NIV: 1/14 at baseline and 1/14 at 2 yr FU	- Supplemental feeding: 1/14 at baseline and 1/14 at 2 yr FU	Sympt *N* = 18	- 16 Nus (median 12 mo) - 2 untreated	NR	- NIV: 3/16 at baseline and 6/16 at 2 yr FU	- Supplemental feeding: 2/16 at baseline and 6/16 at 2 yr FU
Stettner 2023 [21] Switzerland (registry) FU 6–20 mo	Two copies						Sympt *N* = 6 Type 1	- 4 OA (2–6 mo) - 2 Nus + OA (1–3 mo + 3–10 mo)	- CHOP-INTEND mean increase of 28	- 1/6 night ventilation	- 3/6 required nasogastric tube or gastrostomy at end of FU
	Three copies	Presympt *N* = 2 Via FH	- 2 OA (1 mo)	- 2/2 normal motor development; max CHOP INTEND	- 2/2 no respiratory support	- 2/2 no nutritional support	Sympt *N* = 1 Type 2	- 1 OA (17 mo)	- 1/1 reached max CHOP INTEND	- 1/1 no respiratory support	- 1/1 no nutritional support
Schwartz 2024 [22] Germany, Austria, Switzerland (SMArtCARE registry; 70 centres) FU ≥ 18 mo	Two/three copies	Screened *N* = 44 (33 presympt, 11 sympt) *SMN2*: - Two: 31 - Three: 13	- 12 Nus - 7 OA - 21 Nus + OA - 2 Nus + Ris - 2 untreated (mean 1 mo)	Two *SMN2*: 7/31 (23%) asymptomatic at FU Three *SMN2*: 10/13 (77%) asymptomatic at FU	- Baseline: 3/44 (7%) occasional ventilation - After treatment start, 2 (5%) stopped ventilation	- Baseline: 1/44 (2%) supplemental feeding - After treatment start, 1/44 (2%) exclusive tube feeding	Sympt *N* = 190 *SMN2*: One: 1 Two: 110 Three: 79	- 21 Nus - 66 OA - 4 Ris - 72 Nus + OA - 13 Nus + Ris - 5 untreated (mean 11 mo)	NR	- Baseline: 11/190 (6%) perm vent, 19/190 (10%) occasional ventilation - After treatment start, 5/190 (2.6%) started perm vent + 6 (3%) stopped, and 32/190 (17%) occasional ventilation	- Baseline: 14/190 (7%) exclusive tube feeding, 9 (5%) supplemental - After treatment start, 29 (15%) started tube feeding (12 exclusive, 17 supplemental)
Weiss 2022 [23] Germany, Austria (SMArtCARE; 18 centres) FU 6 mo	Two/three copies	Presympt *N* = 6 NR how identified	- 6 OA (NR)	- CHOP INTEND increased from Tx to 6 mo FU (*p* < 0.0001)	NR	NR	Sympt *N* = 50 Type 1 (*N* = 45) Type 2 (*N* = 5)	- 50 OA ± Nus (1–59 mo)	- CHOP INTEND at 6 mo: sig increase in type 1 (*p* = 0.016) but not sig in type 2 (*p* = 0.515)	NR	NR
Servais 2024 [24] (RESTORE; 7 countries, mainly USA) FU 0–37 mo	1/2/3/4 copies	Screened *N* = 20 (presympt or sympt)	- 20 OA (0–72 mo)	- 17/20 CHOP INTEND ≥ 4-point increase - 17/20 had CHOP INTEND ≥ 40 points	NR	NR	Sympt *N* = 21	- 21 OA (0–28 mo)	- 20/21 CHOP INTEND ≥4-point increase - 19/21 had CHOP INTEND ≥ 40 points	NR	NR

Abbreviations: asst, assistance; CHOP INTEND, Children’s Hospital of Philadelphia Infant Test of Neuromuscular Disorders; d, days; FH, family history; FU, follow-up; indep, independently; max, maximum; mo, months; NIV, non-invasive ventilation; NR, not reported; Nus, nusinersen; OA, onasemnogene abeparvovec; presympt, presymptomatic; Ris, risdiplam; sig, significant; SMA, spinal muscular atrophy; *SMN2*, survival motor neuron 2; sympt, symptomatic; Tx, treatment; wk, weeks; yr, years.

**Table 6:** Prospective follow-up of screened cohorts.

Study Follow-Up	Populations Interventions	Survival	Sitting Independently	Standing	Walking	Other Motor and Neurological Outcomes	Respiratory	Feeding
**One *SMN2* copy**								
USA (Georgia) Elkins 2022 [25] FU median 5 mo	One *SMN2* copy - 2 untreated (sympt)	- Untreated: 2/2 died (at 10d and 22 mo)	NR	NR	NR	NR		
USA (New York State) Lee 2022 [26] FU median 12 mo	One *SMN2* copy - 1 Ris at 2 mo (severely sympt)	- Ris: 1/1 alive at FU	NR	NR	NR	- 1 Ris: severe motor symptoms at FU	- Ris: 1/1 ventilator-dependent	- Ris: 1/1 unable to feed orally
**Two *SMN2* copies**								
Belgium Boemer 2021 [27] FU 12–33 mo	Two *SMN2* copies - 4 Nus (3 at 1 mo, 1 at 5 mo) (sympt) - 1 OA at 2 mo (sympt)	- Nus: 4/4 alive - OA: 1/1 alive	- Nus: 3/4 sat indep - OA: 1/1 sat indep	- Nus: NR - OA: 1/1 stood	- Nus: 1/4 walked with asst; 3/4 not walking - OA: 1/1 not walking	- Nus or OA: 5/5 early sympt at treatment; 5/5 developmental delays despite treatment	NR	NR
Germany Vill 2021 [28]; Kolbel 2023 [30]; Schwartz 2022 [31] - FU med 13 mo [Vill]; 10 mo-3.5 yr [Schwartz]	Two *SMN2* copies Vill: - 15 Nus at 0.5–1 mo (8 presympt, 7 sympt) - 2 untreated Schwartz: - 11 Nus at ≤6 wk - 1 OA at ≤6 wk - 9 Nus + OA at ≤6 wk	Vill: - Nus: 15/15 alive - Untreated: 2/2 died Schwartz: - Nus/OA: 21/21 alive	Schwartz: - Nus/OA: 15/21 sat indep in window; 4/21 delayed; 2/21 not met	NR	Schwartz: Nus/OA: - 12/20 walked w asst in window; 5/20 delayed; 3/20 not met - 10/19 walked indep in window; 3/19 delayed; 6/19 not met	Vill: - Nus (presympt): 8/8 met milestones - Nus (sympt, *n* = 7): milestones delayed Schwartz: Nus/OA: - 12/21 met milestones; 3/21 initial delay; 6/21 proximal weakness	Vill: - Nus: 15/15 no respiratory issues - Untreated: 2/2 died (respiratory failure) Schwarz: Nus/OA: - 20/21 no respiratory issues; 1/21 NIV, cough assist	Vill: - Nus: 15/15 no tube feeding - Untreated: NR Schwarz: Nus/OA: - 16/21 no feeding issues; 3/21 mild chewing problems, 2/21 tube feeding
Norway Wallace 2023 [32] (abst) FU NR	Two *SMN2* copies - 5 OA at 0.5 mo (3 sympt, 2 presympt)	- OA (presympt): 2/2 alive - OA (sympt): 2/3 alive	NR	NR	NR	- OA (presympt): 2/2 motor improvements - OA (sympt): 1/3 died, 2/3 NR	NR	NR
USA (California) Matteson 2022 [34] FU to ≥1 yr of age	Two *SMN2* copies - 8 Nus and/or OA (at median 1 mo)	NR	NR	NR	NR	- Nus and/or OA: 6/8 had SMA symptoms at FU (incl 3 presympt); 3 had delays or barriers to Tx	NR	NR
USA (Georgia) Elkins 2022 [25] FU median 5 mo	Two *SMN2* copies - 3 OA at 1–6 mo (2 presympt, 1 sympt) - 2 untreated (sympt)	- OA (presym): 1/1 alive - OA (sympt): 1/1 alive - Untr: 2/2 died	NR	NR	NR	- OA (presympt): 1/1 had symptoms - OA (sympt): 1/1 low CHOP-INTEND - Untreated: 2/2 died	NR	NR
USA (Massachusetts) Hale 2021 [33] FU median 13 mo	Two *SMN2* copies - 1 Nus at 1 mo (presympt) - 4 OA at 0.4–1 mo (1 presympt, 3 sympt) - 1 Nus at 0.4 mo + OA at 3 mo (sympt) - 1 Nus (0.5 mo) + OA (1 mo) + Ris (10 mo) (sympt)	- Treated: 7/7 alive at follow-up	NR	NR	NR	- 1 Nus (presympt): normal - 1 OA (presympt): mild motor delays - 3 OA (sympt): 2 mild-to-mod delays, 1 improved - 1 Nus + OA: normal at FU - 1 Nus + OA + Ris: normal	NR	NR
USA (New York State) Lee 2022 [26] FU median 12 mo	Two *SMN2* copies (10 presym, 8 sympt) - 11 OA at 0.4–2 mo - 1 Nus at 3 mo - 4 Nus (1–2 mo) + OA (2–6 mo) - 2 OA (1 mo) + Ris 6 mo)	- Treated: 18/18 alive at FU	NR	NR	NR	- Treated (presympt): 4/10 symptoms at FU but achieved motor milestones; 6/10 no symptoms - Treated (sympt): 8/8 symptoms at FU	- 10 presympt: no respiratory issues - 8 sympt: 5/8 required NIV	- 10 presympt: no feeding issues - 8 sympt: 3/8 required feeding assistance; no gastrostomy tubes
USA (North Carolina) Kucera 2021 [35] FU 12 wk	Two *SMN2* copies - 1 Nus at 1 mo (sympt)	- Nus: 1/1 alive at 12 wk	NR	NR	NR	- Nus: 1/1 motor symptoms initially worsened but improved by 12 wk	NR	NR
Japan (Hyogo) Noguchi 2022 [36] FU 12 + 24 wk	Two *SMN2* copies - 1 Nus at 1 mo (presympt) - 1 Nus at 1 mo + OA at 4 mo (sympt)	- 2/2 alive at FU	NR	NR	NR	- Nus (presympt): 1/1 little change at 12 wk - Nus + OA (sympt): 1/1 improvements slowed	NR	- 1 Nus (presympt): NR - 1 Nus + OA (sympt): tube feeding
Taiwan Weng 2021 [37] FU median 3 yr	Two *SMN2* copies - 3 Nus at 0.4–3 mo (sympt) - 3 untreated (sympt)	- Nus: 3/3 alive at FU - Untreated: 3/3 died	NR	NR	NR	- Nus: 1/3 walked at 4 yr, 1/3 sitter (supported stand) at 3.3 yr, 1/3 sitter (supported walk) at 1.5 yr - Untreated: 3/3 died	- Nus: 2/3 required ventilatory support - Untreated: NR	- Nus: 2/3 required gastrostomy - Untreated: NR
**Three *SMN2* copies**								
Belgium Boemer 2021 [27] FU 12–33 mo	Three *SMN2* copies - 2 Nus at 1 mo (presympt) - 1 OA at 1 mo (presympt)	- Nus: 2/2 alive - OA: 1/1 alive	- Nus: 2/2 sat indep - OA: 1/1 sat indep	- Nus: 2/2 stood - OA: 1/1 stood	- Nus: 2/2 walked indep - OA: 1/1 walked indep	- Nus or OA: 3/3 hit motor milestones at usual ages	NR	NR
Germany Vill 2021 [28] - FU med 13 mo	Three *SMN2* copies - 6 Nus at 1 mo (presympt) - 4 untreated	- Nus; 6/6 alive - Untreated: 4/4 alive	NR	NR	NR	- Nus (presympt): 5/6 normal motor milestones; 1/6 minimal delay - Untreated: 3/4 proximal weakness; 1/4 motor deterioration	- Nus: 6/6 no respiratory issues - 4 untreated: NR	NR
Norway Wallace 2023 [32] (abst) FU NR	Three *SMN2* copies - 3 OA at 0.5 mo (presympt)	- OA: 3/3 alive	NR	NR	NR	- OA: 3/3 had improvements in motor function, all remained asymptomatic	NR	NR
USA (California) Matteson 2022 [34] FU to ≥1 yr of age	Three *SMN2* copies - 7 Nus and/or OA (at median 1 mo)	NR	NR	NR	NR	- Nus and/or OA: 7/7 no SMA symptoms at FU	NR	
USA (Georgia) Elkins 2022 [25] FU median 5 mo	Three *SMN2* copies - 6 OA at 1–6 mo (presympt) - 1 Nus at 20 mo (sympt)	- OA: 3 alive (3 no data) - Nus: 1/1 alive	NR	NR	NR	- OA: 1/6 some symptoms at 9 mo; 2/6 normal; 3/6 no data - Nus (at 20 mo): 1/1 symptoms progressed; low CHOP-INTEND at 22 mo	NR	
USA (New York State) Lee 2022 [26] FU median 12 mo	Three *SMN2* copies - 10 OA at 0.4–3 mo (presympt) - 1 Nus (1 mo) + OA (NR) (presympt)	- OA/Nus: 11/11 alive	NR	NR	NR	- OA/Nus: 11/11 asymptomatic at FU, meeting milestones	- OA/Nus: 11/11 no respiratory issues	- OA/Nus: 11/11 no feeding issues
Japan (Kumamoto) Sawada 2022 [38] FU 11 mo	Three *SMN2* copies - 1 OA at 1.4 mo (presympt)	- OA: 1/1 alive	NR	NR	NR	- OA: 1/1 normal motor development at 11 mo	NR	NR
Taiwan Weng 2021 [37] FU median 3 yr	Three *SMN2* copies - 3 Nus at 3–6 mo (sympt) - 1 untreated (sympt)	- Nus: 3/3 alive - Untreated: 1/1 alive	NR	NR	NR	- Nus: 1/3 sitter (supported walk) at 2.4 yr, 1/3 walker at 1.3 yr, 1/3 sittter (supported stand) at 0.9 yr - Untreated: 1/1 sitter at 5.3 yr	- Nus: 3/3 no ventilatory support - Untreated: 1/1 required ventilatory support	- Nus: 3/3 no feeding support - Untreated: 1/1 no feeding support
**Four+ *SMN2* copies**								
Belgium Boemer 2021 [27] FU 12–33 mo	Four+ *SMN2* copies - 1 Nus at 2 mo (presympt) - 1 Ris at 1 mo (presympt)	- Nus: 1/1 alive - Ris: 1/1 alive	- Nus: 1/1 sat indep - Ris: 1/1 sat indep	- Nus: 1/1 stood - Ris: 1/1 stood	- Nus: 1/1 walked indep - Ris: 1/1 walked indep	- Nus or Ris: 2/2 asymptomatic at treatment; 2/2 hit motor milestones at usual ages	NR	NR
Germany Vill 2021 [28]; Blaschek 22 [29] - FU med 13 mo [Vill] - FU 10 mo-3.5 y [Schwartz]	Four+ *SMN2* copies - 8 treated at 3–36 mo (presympt) - 7 untreated (presympt)	- Treated: 8/8 alive - Untreated: 7/7 alive	NR	NR	NR	- Treated: 8/8 asymptomatic - Untreated: 5/7 symptomatic (at 1.5 to 4 yr); 2 of 5 no complete recovery despite symptomatic treatment	NR	NR
Norway Wallace 2023 [32] (abst) FU NR	Four+ *SMN2* copies - 2 OA (timing NR) (presympt)	- OA: 2/2 alive	NR	NR	NR	- OA: 2/2 improvements in motor function	NR	NR
USA (California) Matteson 2022 [34] FU to ≥1 yr of age	Four+ *SMN2* copies - 1 Nus and/or OA (at median 2 mo)	NR	NR	NR	NR	- Nus/OA: 1/1 had SMA symptoms at FU	NR	NR
USA (Georgia) Elkins 2022 [25] FU median 5 mo	Four+ *SMN2* copies - 2 untreated (presympt)	- Untreated: 1 alive (1 no data)	NR	NR	NR	- Untreated: 1 normal exam at 1.5 mo (1 no data)	NR	NR
USA (Massachusetts) Hale 2021 [33] FU median 13 mo	Four+ *SMN2* copies - 1 Nus at 0.3 mo (presympt) - 1 OA at 6 mo (sympt)	- Nus/OA: 2/2 alive at follow-up	NR	NR	NR	- 1 Nus (presympt): no symptoms at FU - 1 OA (sympt): no symptoms at FU	NR	NR
USA (New York State) Lee 2022 [26] FU median 12 mo	Four+ *SMN2* copies - 2 OA at 6 mo (presympt) - 2 untreated (presympt)	- OA: 2/2 alive - Untreated: 2/2 alive	NR	NR	NR	- OA: 2/2 asymptomatic at FU - Untreated: 2/2 asymptomatic at FU	- OA: 2/2 no respiratory issues - Untreated: 22 no respiratory issues	- OA: 2/2 no feeding issues - Untreated: 22 no feeding issues
7 countries, mainly USA (RESTORE) Finkel 2023 [39] (abst) FU mean 14 mo	Four+ *SMN2* copies - 19 OA at 1–11 mo	- OA: 19/19 alive at FU	NR	NR	NR	- OA: Of 12 with data, 12/12 achieved new motor milestones - OA: Of 13 with data, 7/13 CHOP INTEND max (64)	- OA (*n* = 19): No respiratory support	- OA (*n* = 19): No nutritional support
Taiwan Weng 2021 [37] FU median 3 yr	Four+ *SMN2* copies - 4 untreated (3 presympt, 1 sympt)	- Untreated: 4/4 alive at FU	NR	NR	NR	- Untreated (presympt): 3/3 no symptoms at FU - Untreated (sympt): 1/1 walker at 3.4 yr	- Untreated: 4/4 no ventilatory support	- Untreated: 4/4 no feeding support

Abbreviations: abst, abstract; asst, assistance; CHOP INTEND, Children’s Hospital of Philadelphia Infant Test of Neuromuscular Disorders; d, days; FU, follow-up; indep, independently; mo, months; NIV, non-invasive ventilation; NR, not reported; Nus, nusinersen; OA, onasemnogene abeparvovec; presympt, presymptomatic; Ris, risdiplam; SMA, spinal muscular atrophy; *SMN2*, survival motor neuron 2; sympt, symptomatic; untr, untreated; wk, weeks; yr, years.

**Table 7:** Risk of bias in included studies.

Study Ref(s)	Population: Representative Cohort? ^a^	Intervention: Fidelity to Intended Intervention?	Outcomes: Blinded Outcome Assessment?	Outcomes: Sufficient Follow-Up? (≥1 yr)	Outcomes: At Least 90% Analysed?
**Single-arm interventional studies**					
NURTURE; Crawford 2023 [10,11,12]	U	Y	N	Y	Y (25/25 = 100%)
SPR1NT; Strauss 2022 [7,15,16,17]	U	Y	N	Y	Y (29/29 = 100%)
RAINBOWFISH; Finkel 2022 [13]	U	Y	N	Y	N (7/18 = 39%)
**Prospective comparative studies**					
Australia; Kariyawasam 2023 [18,19]	Y	U	N	Y	Y (33/33 = 100%)
Belgium; Ngawa 2023 [20]	Y	U	N	Y	Y (18/18 = 100%)
Switzerland; Stettner 2023 [21]	Y	U	N	N	Y (9/9 = 100%)
Schwartz 2024 [22]	Y	U	N	Y	Y (234/234 = 100%)
Germany + Austria; Weiss 2022 [23]	Y	U	N	N	N (56/76 = 74%)
7 countries (RESTORE); Servais 2024 [24]	Y	U	N	Y	N (41/168 = 24%)
**Screening studies with follow-up**					
Belgium; Boemer 2021 [20,27,40]	Y	U	N	Y	Y (10/10 = 100%)
Germany; Vill 2021 [28,29,30,31]	Y	U	N	Y	Y (43/43 = 100%)
Norway; Wallace 2023 [32] (abst)	Y	U	N	U	Y (10/10 = 100%)
USA (California); Matteson 2022 [34]	Y	U	N	Y	N (16/34 = 47%)
USA (Georgia); Elkins 2022 [25]	Y	U	N	N	N (11/16 = 69%)
USA (Massachusetts); Hale 2021 [33]	Y	U	N	Y	Y (9/9 = 100%)
USA (New York State); Lee 2022 [26]	Y	U	N	Y	Y (34/34 = 100%)
USA (North Carolina); Kucera 2021 [35]	Y	U	N	N	Y (1/1 = 100%)
7 countries (RESTORE); Finkel 2023 [39]	Y	U	N	Y	N (12/19 = 63%)
Japan (Kumamoto); Sawada 2022 [38]	Y	U	N	N	Y (1/1 = 100%)
Japan (Hyogo); Noguchi 2022 [36]	Y	U	N	N	Y (2/2 = 100%)
Taiwan; Weng 2021 [37]	Y	U	N	Y	N (14/21 = 67%)

^a^ Considered representative cohort if presymptomatic SMA and either interventional study or consecutive or random cohort. Abbreviations: abst, abstract; yr, years.

**Table 8:** Adverse events.

**Nusinersen: NURTURE** Crawford 2023 [10]	**Risdiplam: RAINBOWFISH** Finkel 2022 [13,14]	**Onasemnogene abeparvovec: SPR1NT** Strauss 2022 [7]; Strauss 2022 [15]	**Onasemnogene abeparvovec: screening** RESTORE registry [24]; Swiss study [21]
Special warnings and precautions [41,42] - Risk of adverse effects of lumbar puncture - Thrombocytopenia and coagulation abnormalities have been observed with other antisense oligonucleotides - Renal toxicity has been observed with other antisense oligonucleotides - Hydrocephalus after nusinersen has been reported in the post-marketing setting	Special warnings and precautions [43,44] - Embryo–foetal toxicity has been observed in animal studies - Effects on male fertility have been observed in animal studies - Retinal toxicity has been observed in non-clinical safety studies, but not in clinical studies in SMA patients	Special warnings and precautions [45,46] - Hepatotoxicity: cases of acute liver failure with fatal outcomes; acute serious liver injury and elevated aminotransferases; may be immune-mediated; corticosteroid recommended - Systemic immune response: possible risk for patients with underlying infection - Thrombocytopenia: transient decreases in platelet counts frequently observed - Thrombotic microangiopathy: cases observed - Cardiac effects: increases in cardiac troponin-I levels observed in clinical trials, and cardiac toxicity in animal studies - Risk of tumorigenicity (theoretical) due to integration of AAV vector into genome	See left
AE summary (NURTURE) [10] - Any AE: 25/25 (100%) - Moderate or severe AE: 19/25 (76%) - Severe AE: 6/25 (24%) - Serious AE: 12/25 (48%) - AE leading to discontinuation of drug/study: 0 - AE, considered study-drug related by investigators: 0 (0%) - AE, considered possibly study-drug related by investigators: 10/25 (40%) - Serious AE, considered study-drug-related by investigators: 0 (0%) - AE related or possibly related to lumbar puncture: 13/25 (52%) - All resolved despite continued treatment, except for proteinuria (*n* = 1) and clonus (*n* = 1)	AE summary (RAINBOWFISH) [13] - At least one AE: 14/18 (78%) - AEs considered treatment-related: 2/18 (11%) (1 diarrhoea, 1 skin discoloration) - Grade 3–5 AEs: 2/18 (11%), both Gd3, neither considered treatment-related (1 gastroenteritis norovirus; 1 cystoid macular oedema) - Serious AEs: 0/18 (0%) [Finkel 2022] - Serious AEs: 8/26 (31%) [ClinicalTrials.gov] [14] - Deaths: 0 (0%) - AEs leading to treatment withdrawal: 0 (0%) - AEs leading to dose modification or interruption: 2/18 (11%), neither considered treatment-related (AE type not specified)	AE summary (SPR1NT, two + three copy cohorts) [7,15] - AEs: 14/14 (100%); 15/15 (100%) - Treatment-related AEs: 10/14 (71%); 8/15 (53%) - Serious AEs: 5/14 (36%); 3/15 (20%) - Treatment-related serious AEs: 0 (0%); 0 (0%) - To attenuate the inflammatory response, all patients commenced oral prednisolone 1 day before infusion and completed a median of 60 days [two *SMN2* copy cohort] or 63 days [three copy cohort]	AE summary (RESTORE registry; screened + symptomatic cohorts) [24] (screened cohort; symptomatic cohort) - Any AE: 35/97 (36%); 46/70 (66%) - Grade 3+ AE: 11/97 (11%); 29/70 (41%) - Serious AE: 9/97 (9%); 22/70 (31%) - Treatment-related AE: 26/97 (27%); 28/70 (40%) - Serious treatment-related AE: 4/97 (4%); 4/70 (6%)
-	Serious AEs (RAINBOWFISH) [14] - Urinary tract infection: 2/26 (8%) - Gastroenteritis: 2/26 (8%) - Constipation: 1/26 (4%) - Femur fracture: 1/26 (4%) - Soft tissue injury: 1/26 (4%) - Jaundice, neonatal: 1/26 (4%)	AEs of special interest (SPR1NT, two + three copy cohorts) [7,15] - Hepatotoxicity: 3/14 (21%); 4/15 (27%) - Thrombocytopenia: 3/14 (21%); 2/15 (13%) - Cardiac AEs: 2/14 (14%); 3/15 (20%) - Thrombotic microangiopathy: 2/14 (14%); 0/15 (0%) - Sensory abnormalities suggestive of dorsal root ganglionopathy: 3/14 (21%); 1/15 (7%)	AEs of special interest (RESTORE registry; screened + symptomatic cohorts) [24] - Hepatotoxicity: 19/97 (20%); 30/70 (43%) - Transient thrombocytopenia: 5/97 (5%); 18/70 (26%) - Cardiac AEs: 8/97 (8%); 14/70 (20%) - Thrombotic microangiopathy: 0/97 (0%); 1/70 (1.4%)
AEs, potentially treatment-related (NURTURE) [10] - ALT increased, AST increased, eosinophil count increased, lymphocyte count increased, WBC count increased, pyrexia: 1/25 (4%) - Blood alkaline phosphatase increased, blood calcium increased, protein urine present: 1/25 (4%) - Clonus, extensor plantar response, muscular weakness, weight-bearing difficulty: 1/25 (4%) - Dermatitis, allergic: 1/25 (4%) - Headache: 1/25 (4%) - Hyperreflexia, tachycardia: 1/25 (4%) - Platelet count increased: 1/25 (4%) - Protein urine present: 1/25 (4%) - Proteinuria: 1/25 (4%) - Rash: 1/25 (4%)	-	AEs, potentially treatment-related (SPR1NT, two + three copy cohorts) [7,15] - Gastrointestinal disorders: 5/14 (36%); 3/15 (20%) - Aspartate aminotransferase increased: 3/14 (21%); 4/15 (27%) - Rash or skin disorders: 2/14 (14%); 3/15 (20%) - Alanine aminotransferase increased: 1/14 (7%); 3/15 (20%) - Blood creatinine phosphokinase MB increased: 1/14 (7%); 2/15 (13%) - Troponin increased: 1/14 (7%); 2/15 (13%) - Gamma-glutamyl transferase increased: 1/14 (7%); 1/15 (7%) - Platelet count increased: 1/14 (7%); 1/15 (7%) - Blood creatinine phosphokinase increased: 1/14 (7%); NR - Blood alkaline phosphatase increased: NR; 1/15 (7%) - Platelet count decreased: 1/14 (7%); NR - Thrombocytopenia: 1/14 (7%); NR - Eye discharge: 1/14 (7%); NR - Malaise: 1/14 (7%); NR - Motor development delay: 1/14 (7%); NR - Feeding or weight gain poor: NR; 2/15 (13%) - Agitation: NR; 1/15 (7%) - Cough: NR; 1/15 (7%) - Iron deficiency anaemia: NR; 1/15 (7%) - Cushingoid: NR; 1/15 (7%) - Pyrexia: NR; 1/15 (7%) - Nasopharyngitis: NR; 1/15 (7%)	-
Most common AEs (in ≥5 infants) (NURTURE) [10] - Pyrexia: 21/25 (84%) - Upper respiratory tract infection: 18/25 (72%) - Cough: 16/25 (64%) - Nasopharyngitis: 16/25 (64%) - Vomiting: 12/25 (48%) - Rhinorrhea: 11/25 (44%) - Fall: 10/25 (40%) - Muscular weakness: 10/25 (40%) - Diarrhoea: 9/25 (36%) - Influenza: 9/25 (36%) - Nasal congestion: 8/25 (32%) - Otitis media: 8/25 (32%) - Tremor: 8/25 (32%) - Constipation: 7/25 (28%) - Pneumonia: 7/25 (28%) - Seasonal allergy: 7/25 (28%) - Anaemia: 6/25 (24%) - Dehydration: 6/25 (24%) - Gait disturbance: 6/25 (24%) - Gastroenteritis, viral: 6/25 (24%) - Dermatitis, diaper: 5/25 (20%) - Ear infection: 5/25 (20%) - Respiratory tract infection: 5/25 (20%) - Rhinitis: 5/25 (20%) - Speech disorder, developmental: 5/25 (20%) - Tachycardia: 5/25 (20%)	Most common AEs (in ≥3 infants) (RAINBOWFISH) [13] - Teething: 6/18 (33%) - Nasal congestion: 5/18 (28%) - Pyrexia: 5/18 (28%) - Diarrhoea: 4/18 (22%) - Viral infection: 4/18 (22%) - Vomiting: 4/18 (22%) - Constipation: 3/18 (17%) - Cough: 3/18 (17%) - Eczema: 3/18 (17%)	Most common AEs (in ≥2 infants) (SPR1NT, two + three copy cohorts) [7,15] - Pyrexia: 7/14 (50%); 11/15 (73%) - Upper respiratory tract infection: 5/14 (36%); 9/15 (60)% - Aspartate aminotransferase increased: 3/14 (21%); 4/15 (27%) - Diarrhoea: 3/14 (21%); 4/15 (27%) - Teething: 2/14 (14%); 5/15 (33%) - Gastroesophageal reflux disease: 3/14 (21%); 3/15 (20%) - Rash: 3/14 (21%); 2/15 (13%) - Nasal congestion: 3/14 (21%); 2/15 (13%) - Hypotonia: 3/14 (21%); 2/15 (13%) - Vomiting: 3/14 (21%); 2/15 (13%) - Nasopharyngitis: 2/14 (14%); 3/15 (20%) - Constipation: 4/14 (29%); NR - Cough: NR; 4/15 (27%) - Viral upper respiratory tract infection: 3/14 (21%); NR - Tremor: 3/14 (21%); NR - Muscle contractions, involuntary: 3/14 (21%); NR - Dermatitis, diaper: NR; 3/15 (20%) - Alanine aminotransferase increased: NR; 3/15 (20%) - Otitis media: NR; 3/15 (20%) - Ear infection: 2/14 (14%); NR - Areflexia: 2/14 (14%); NR - Eczema: 2/14 (14%); NR - Influenza: 2/14 (14%); NR - Rhinovirus infection: 2/14 (14%); NR - Blood calcium increased: NR; 2/15 (13%) - Blood creatinine phosphate MB increased: NR; 2/15 (13%) - Microcytic anaemia: NR; 2/15 (13%) - Gastroenteritis: NR; 2/15 (13%) - Hand-foot-and-mouth disease: NR; 2/15 (13%) - Troponin increased: NR; 2/15 (13%) - Urinary tract infection: NR; 2/15 (13%)	Swiss cohort [21] (presymptomatic patients with OA and three *SMN2* copies; *n* = 9) - 100% transient decrease of platelet count - 3/9 (33%) thrombocytopenia - 100% transaminase increase - Troponin-T elevated prior to OA in 100% and showed fluctuations in 57% thereafter

Abbreviations: AE, adverse event; AESI, adverse event of special interest; OA, onasemnogene abeparvovec; SMA, spinal muscular atrophy; *SMN2*, survival motor neuron 2.

## Appendix A. Medline Search Strategy

1. exp “Spinal Muscular Atrophies of Childhood”/
2. exp Muscular Atrophy, Spinal/
3. (werdnig-hoffman or werdnig hoffman).tw.
4. (kugelberg-welander or kugelberg welander).tw.
5. Spinal muscular atroph*.tw.
6. or/1–5
7. randomized controlled trial.pt. or randomized.mp. or placebo.mp.
8. (“clinical trial” or “clinical trial, phase i” or “clinical trial, phase ii” or clinical trial, phase iii or clinical trial, phase iv or controlled clinical trial or “multicenter study” or “randomized controlled trial”).pt. or double-blind method/ or clinical trials as topic/ or clinical trials, phase i as topic/ or clinical trials, phase ii as topic/ or clinical trials, phase iii as topic/ or clinical trials, phase iv as topic/ or controlled clinical trials as topic/ or randomized controlled trials as topic/ or early termination of clinical trials as topic/ or multicenter studies as topic/ or ((randomi?ed adj7 trial*) or (controlled adj3 trial*) or (clinical adj2 trial*) or ((single or doubl* or tripl* or treb*) and (blind* or mask*))).ti,ab,kw. or (“4 arm” or “four arm”).ti,ab,kw.
9. 7 or 8
10. 6 and 9
11. Epidemiologic studies/
12. exp case control studies/
13. exp cohort studies/
14. Case control.tw.
15. (cohort adj (study or studies)).tw.
16. Cohort analy$.tw.
17. (Follow up adj (study or studies)).tw.
18. (observational adj (study or studies)).tw.
19. Longitudinal.tw.
20. Retrospective.tw.
21. Cross sectional.tw.
22. Cross-sectional studies/
23. or/11–22
24. 6 and 23
25. meta analysis.mp,pt. or review.pt. or search:.tw.
26. 6 and 25
27. 10 or 24 or 26
